# COVID-19 in Iran: clinical presentations and outcomes in three different surges of COVID-19 infection

**DOI:** 10.1186/s12985-022-01846-7

**Published:** 2022-07-26

**Authors:** Azar Hadadi, Marzieh Pirzadeh, Sina Kazemian, Haleh Ashraf, Mehdi Ebrahimi, Shahrokh Karbalai Saleh, Mohammad Talebpour

**Affiliations:** 1 Department of Infectious Diseases, Sina Hospital, Tehran University of Medical Sciences, Tehran, Iran; 2 Research Development Center, Sina Hospital, Tehran University of Medical Sciences, Tehran, Iran; 3 Students’ Scientific Research Center (SSRC), Tehran University of Medical Sciences, Tehran, Iran; 4 Cardiac Primary Prevention Research Center (CPPRC), Cardiovascular Diseases Research Institute, Tehran University of Medical Sciences, Tehran, Iran; 5 Department of Endocrinology Medicine, Sina Hospital, Tehran University of Medical Sciences, Hasan Abad Sq, Tehran, Iran; 6 Department of Cardiology, Sina Hospital, Tehran University of Medical Sciences, Tehran, Iran; 7 Department of Surgery, Sina Hospital, Tehran University of Medical Sciences, Hasan Abad Sq., Tehran, 11367-46911 Iran

**Keywords:** ARDS, COVID-19, Comorbidities, Mortality

## Abstract

**Background:**

A few studies compared the characteristics and outcomes of COVID-19 patients during the first and second surges of the disease. We aimed to describe the clinical features and outcomes of COVID-19 patients across the first, second, and third surges of the disease in Tehran, Iran.

**Method:**

We conducted a retrospective cohort study of patients with COVID-19 admitted to Sina hospital in Tehran, Iran, during three surges of COVID-19 from February 16 to October 28, 2020.

**Result:**

Surge 1 patients were younger with more prevalence of hypertension. They also presented with significantly higher oxygen saturation, systolic blood pressure, and respiratory rate on admission. Patients had higher levels of neutrophil to lymphocyte ratio, Urea, CRP, and ESR, in surge 2. The incidence of dyspnea, chest pain, and neurological manifestations followed a significant increasing trend from surge 1 to surge 3. There was no difference in severity and in-hospital mortality between the surges. However, the length of hospital stays and acute cardiac injury (ACI) was less in surge 1 and acute respiratory distress syndrome (ARDS) in surge 2 than in other surges.

**Conclusion:**

Patients did not significantly differ in disease severity, ICU admission, and mortality between surges; however, length of hospital stay and ACI increased during surges, and the number of patients developing ARDS was significantly less in surge 2 compared to other peaks.

## Introduction

Iran has been one of the most affected countries during the COVID-19 pandemic in the middle east, experiencing the COVID-19 outbreak with 6,019,947 confirmed cases and 127,809 deceased cases till November 12, 2021 [1]. During some periods of COVID-19 outbreaks, Iran was ranked the third and second country worldwide regarding the total number of patients and mortality, respectively [2]. Up to now, this country has faced different surges of COVID-19 outbreak. Previous observations have reported COVID-19 mortality association with several risk factors and comorbidities such as diabetes, hypertension, cancer, cardiovascular disease, chronic kidney disease, and other chronic diseases [3, 4]. Thus, evaluating the epidemiological characteristics of COVID-19 and specifying the underlying comorbidities of SARS-CoV2 infected patients could be of great importance to help public health officials, decision-makers, and clinicians to make pragmatic strides toward reducing the burden of COVID-19 and the subsequent control of the pandemic during upcoming surges.

This study aims to describe the demographic features, comprehensive clinical and laboratory parameters, and in-hospital outcome differences across the first, second, and third surges of COVID-19 hospitalized patients during a large cohort study in Iran.

## Methods

All participants provided informed written consent before enrolling in the study, and the ethics committee of the Tehran University of Medical Sciences was approved for the study (IR.TUMS.VCR.REC.1399.018.). Our study has been performed in accordance with the declaration of Helsinki.

This retrospective cohort study was performed in the Sina Hospital, a COVID-19 referral center affiliated with Tehran University of Medical Sciences (TUMS) in Tehran, Iran, From February 16 to October 28, 2020. We included patients over 18 years of age with the diagnosis of COVID-19 who fulfilled one of the two following criteria: (i) PCR -confirmed Covid-19 oropharyngeal or endotracheal swab specimens. (ii) Highly suspected COVID-19 patients according to the World Health Organization's interim guidance [5] and Iranian national committee of COVID-19 [6], including patients with the signs of COVID-19 involvement in their chest computed tomography scan (presence of ground-glass opacity, either isolated or with consolidation), which cannot be fully defined by volume overload, lobar or lung collapse, or nodules along with the history compatible with COVID-19. Demographic data, comorbidities, clinical presentations, vital signs on admission, and laboratory data were extracted from patients' electronic medical records. All patients were followed up for in-hospital complications, severe COVID-19 manifestations, and in-hospital mortality across their admission period. We classified patients into three surges based on admission time: surge 1 (February 15 to April 30, 2020), surge 2 (May 1 to August 30, 2020), and surge 3 (September 1 to October 30, 2020). Disease severity, comorbidities, and in-hospital outcomes were defined based on previous studies [7, 8].

Categorical variables were presented as numbers (%) and compared using the chi-square and chi-square post hoc test, in which we assumed *P* ≤ 0.00833 as statistically significant according to the Bonferroni correction. The normality of distribution for numerical variables was evaluated by Kolmogorov–Smirnov and Shapiro–Wilk tests. Numerical variables with normal distribution were presented as mean ± standard deviation and compared using the one-way ANOVA test. In contrast, variables with skewed distribution were presented as median [interquartile range] and compared using the Kruskal–Wallis test. All statistical analyses were performed using SPSS Statistics for Windows, Version 21, and *P* < 0.05 was considered statistically significant.

## Results

### Demographic and baseline characteristics

As of February 16 to October 28, 2020, 19,722 patients were screened, and 3309 patients were admitted with COVID-19 diagnosis. A total of 649 (19.6%) patients were deceased during this cohort, of whom 387 (59.6%), 149 (23.0%), and 113 (17.4%) patients were admitted to the intensive care unit (ICU), emergency department, and wards respectively. The majority of deceased patients (160 out of 649 (24.6%)) were in the 70–80 age group. In this study, we excluded patients with a lack of key information in their medical records. Finally, 1323 patients were included representing 667, 489, and 167 patients during surges 1, 2, and 3. Baseline characteristics of patients during the three surges are presented in Table. 1. During the first, second, and third surges, the mean ages of hospitalized patients were 57.6 ± 16.3, 61.0 ± 16.6, and 60.7 ± 16.6, respectively (*P*: 0.001). Comparing the three waves, men constituted the majority of COVID-19 cases (61.1%) during our study period; however, patients did not differ between the three surges with similar gender and body mass index (BMI). Hypertension was the most common comorbidity presenting in 595 patients (45.0%) in all three surges, followed by diabetes mellitus (29.7%) and a history of cardiac disease (23.3%). The prevalence of comorbidities did not differ significantly between the surges except for hypertension (*P*: 0.015), and the proportion of patients with hypertension was significantly lower in surge 1 in comparison with surges 2 and 3 (*P* < 0.0083).

**Table 1:** Baseline characteristics and in-hospital outcomes of COVID-19 during different surges

Characteristic†		Total (N = 1323)	Surge 1 (N = 667)	Surge 2 (N = 489)	Surge 3 (N = 167)	P*
*Demographics*						
Age (year)		59.2 ± 16.5	57.6 ± 16.3	61.0 ± 16.6	60.7 ± 16.6	**0.001**
*Sex*						
Female		515(38.9%)	249(37.3%)	197(40.3%)	69(41.3%)	0.473
Male		808(61.1%)	418(62.7%)	292(59.7%)	98(58.7%)	
BMI (kg/m^2^)		27.4 ± 4.7	27.5 ± 4.9	27.3 ± 4.6	27.4 ± 4.6	0.917
*Comorbidities*						
Hypertension		595(45.0%)	274(41.1%)**	237(48.5%)	84(50.3%)	**0.015**
Diabetes mellitus		393(29.7%)	197(29.5%)	145(29.7%)	51(30.5%)	0.968
Cardiac disease		308(23.3%)	147(22.0%)	122(24.9%)	39(23.4%)	0.512
Cerebrovascular disease		58(4.4%)	22(3.3%)	28(5.7%)	8(4.8%)	0.133
Chronic lung disease		82(6.2%)	50(7.5%)	22(4.5%)	10(6.0%)	0.112
Malignancy		59(4.5%)	28(4.2%)	25(5.1%)	6(3.6%)	0.641
Chronic kidney disease		63(4.8%)	33(4.9%)	23(4.7%)	7(4.2%)	0.971
*Symptoms*						
Fever		721(54.5%)	354(53.1%)	271(55.4%)	96(57.5%)	0.518
Cough		825(62.4%)	447(67.0%)**	273(55.8%)**	105(62.9%)	**0.001**
Dyspnea		799(60.4%)	361(54.1%)**	325(66.5%)**	113(67.7%)	** < 0.001**
Myalgia and arthralgia		637(48.1%)	273(40.9%)**	261(53.4%)**	103(61.7%)**	** < 0.001**
Headache		223(16.9%)	100(15.0%)	89(18.2%)	34(20.4%)	0.154
Nausea and vomiting		354(26.8%)	133(19.9%)**	151(30.9%)**	70(41.9%)**	** < 0.001**
Sore throat		57(4.3%)	31(4.6%)	16(3.3%)	10(6.0%)	0.272
Chest pain		167(12.6%)	62(9.3%)**	70(14.3%)	35(21.0%)**	** < 0.001**
Abdominal pain		227(17.2%)	160(24.0%)**	44(9.0%)**	23(13.8%)	** < 0.001**
Neurological manifestations		110(8.3%)	38(5.7%)**	42(8.6%)	30(18.0%)**	** < 0.001**
LOC		83(6.3%)	35(5.2%)	35(7.2%)	13(7.8%)	0.288
*Vital signs on admission*						
Heart rate		91.2 ± 24.0	88.6 ± 16.4	93.7 ± 32.5	93.4 ± 15.5	** < 0.001**
SBP (mmHg)		126.3 ± 21.2	123.8 ± 20.5**	128.3 ± 21.8	128.7 ± 21.4	** < 0.001**
DBP (mmHg)		76.6 ± 12.9	75.8 ± 11.7	76.9 ± 13.4	78.2 ± 15.0	0.131
Respiratory rate		21.0 ± 5.7	19.8 ± 5.6**	22.3 ± 5.9	21.2 ± 4.6	** < 0.001**
Temperature (^o^C)		37.2 ± 0.9	37.2 ± 0.9	37.2 ± 0.9	37.1 ± 0.9	0.706
Oxygen saturation (%)		89.2 ± 8.1	90.7 ± 7.4**	87.7 ± 8.8	88.1 ± 7.7	** < 0.001**
*Laboratory data on admission*						
WBC (× 10^9^/L)		6.9[5.2–9.5]	6.6[5.2–9.5]	7.4[5.3–9.5]	7.2[5.1–9.6]	0.338
Neutrophil (× 10^9^/L)		5.3[3.7–7.7]	4.8[3.6–7.3]	5.9[3.9–8.1]	5.6[3.7–7.5]	**0.003**
Lymphocyte (× 10^9^/L)		1.1[0.8–1.6]	1.2[0.9–1.7]	1.0 [0.7–1.4]	1.1[0.8–1.5]	** < 0.001**
Platelets (× 10^9^/L)		194.0[151.0–265.5]	191.0[150.0–258.0]	203.0[157.0–282.7]	192.5[152.0–262.2]	0.094
Neutrophil-to-lymphocyte ratio		4.5[2.7–8.1]	3.8[2.5–6.5]	5.9[3.4–10.1]	4.7[3.0–7.0]	** < 0.001**
Platelet-to-lymphocyte ratio		173.1[119.7–259.3]	154.4[114.0–216.1]	206.8[138.3–336.1]**	178.1[121.7–259.9]	** < 0.001**
SII		889.6[498.5–1745.4]	762.1[445.8–1380.9]	1221.4[610.2–2607.7]**	913.2[518.1–1714.6]	** < 0.001**
Hemoglobin (g/dL)		13.5[12.1–14.9]	13.7[12.4–15.0]	13.3[11.8–14.6]	13.5[12.0–14.8]	**0.008**
Urea (mg/dL)		34.0[24.0–53.0]	32.0[23.0–49.0]	39.0[27.0–60.0]**	34.0[25.7–48.0]	** < 0.001**
BUN/creatinine ratio		14.7[11.2–19.5]	13.9[10.7–18.3]	15.9[12.2–20.7]	14.7[11.3–18.6]	** < 0.001**
Creatinine (mg/dL)		1.1[0.9–1.3]	1.1[0.9–1.3]	1.1[0.9–1.3]	1.1[0.9–1.3]	0.157
Sodium (mmol/L)		136.3[133.1–139.4]	136.0[132.8–139.4]	137.0[133.8–139.9]	135.9[132.7–138.2]	**0.009**
Potassium (mmol/L)		4.3[4.0–4.7]	4.3[3.4–4.6]	4.4[4.0–4.9]	4.4[3.9–4.7]	** < 0.001**
Calcium (mmol/L)		8.8[8.3–9.2]	8.7[8.2–9.1]**	8.8[8.4–9.2]	8.8[8.4–9.2]	** < 0.001**
Phosphorous (mmol/L)		3.3[2.8–3.9]	3.4[2.9–4.0]	3.3[2.7–4.0]	3.0[2.5–3.6]	** < 0.001**
Magnesium (mmol/L)		2.2[2.0–2.5]	2.2[2.0–2.5]	2.1[1.9–2.4]	2.2[1.9–2.6]	** < 0.001**
CRP (mg/L)		62.1[27.1–99.5]	56.0[19.8–98.3]	81.4[37.4–99.5]	49.5[28.8–111.8]	**0.001**
ESR (mm/h)		50.0[28.0–81.0]	44.0[26.0–75.0]**	56.0[36.0–87.0]	55.5[35.0–87.2]	** < 0.001**
LDH (U/L)		603.5[472.7–793.0]	536.0[435.0–700.0]**	648.0[513.0–822.0]	713.5[532.5–925.0]	** < 0.001**
hs-cTnI (pg/mL)		5.3[1.5–17.8]	6.0[1.5–19.8]	5.2[1.5–16.1]	4.4[1.5–16.4]	0.650
AST (U/L)		53.0[39.7–71.0]	50.0[38.0–67.7]	56.0[42.0–71.2]	55.5[41.0–76.7]	**0.010**
ALT (U/L)		39.0[29.0–56.0]	36.0[27.0–51.0]	43.0[31.0–61.2]	39.0[28.0–57.0]	** < 0.001**
ALP (U/L)		175.0[137.0–232.0]	171.0[131.0–224.0]	175.5[142.7–237.0]	180.5[137.2–425.5]	0.108
*In-hospital outcomes*						
Hospital length of stay (day)		5.0[2.2–8.0]	4.0[2.0–7.0]**	6.0[3.0–9.0]	6.0[4.0–9.0]	** < 0.001**
ICU admission		217(16.4%)	111(16.6%)	81(16.6%)	25(15.0%)	0.866
Severity		896(67.7%)	437(65.5%)	349(71.4%)	110(65.9%)	0.094
Mortality		235(17.8%)	114(17.1%)	91(18.6%)	30(18.0%)	0.798
ARDS		373(28.2%)	205(30.7%)	46(23.7%)**	52(31.1%)	**0.022**
Invasive ventilation		169(12.8%)	97(14.5%)	53(10.8%)	19(11.4%)	0.149
ACI		303(22.9%)	133(19.9%)	121(24.7%)	49(29.3%)	**0.017**
AKI		173(13.1%)	83(12.4%)	65(13.3%)	25(15.0%)	0.676
ALI		147(11.1%)	64(9.6%)	62(12.7%)	21(12.6%)	0.209
Multi-organ damage		260(19.7%)	127(19.0%)	95(19.4%)	38(22.8%)	0.551

*ACI* acute cardiac injury, *AKI* acute kidney injury, *ALI* acute liver injury, *ALP* alkaline phosphatase, *ALT* alanine transaminase, *ARDS* acute respiratory distress syndrome, *AST* aspartate aminotransferase, *BMI* body mass index, *BUN* blood urea nitrogen, *CRP* C-reactive protein, *DBP* diastolic blood pressure, *ESR* erythrocyte sedimentation rate, *hs-cTnI* high sensitive cardiac troponin I, *ICU* intensive care unit, *LDH* lactate dehydrogenase, *LOC* loss of consciousness, *SBP* systolic blood pressure, *SII* systemic immune-inflammation index, *WBC* white blood cells

^†^Data are presented as mean ± standard deviation, number (%), or median [interquartile range]

^*^Statistically significant *P*-values are bolded

^**^Statistically significant post hoc Bonferroni's *P*-value (*P*-value < 0.0083)

### Clinical presentation

Clinical presentation symptoms including cough, dyspnea, fever, myalgia, and arthralgia were the most common among hospitalized patients with COVID-19. Apart from fever, headache, sore throat, and loss of consciousness, the prevalence of all symptoms differed significantly between the surges (*P* < 0.001 except for cough *P*: 0.001). After Bonferroni's post hoc analysis, we found out that the incidence of dyspnea, myalgia and arthralgia, nausea and vomiting, chest pain, and neurological manifestations followed a significant increasing trend from surge 1 to surge 3 (*P* < 0.008). Cough and abdominal pain were more frequent in surge 1 and then significantly decreased during surge 2.

### Clinical and laboratory findings

The vital signs and laboratory parameters on admission are presented in Table.1. The heart rate, systolic blood pressure (SBP), respiratory rate, and oxygen saturation of patients were significantly different across the surges (*P* < 0.001). Application of the Bonferroni post hoc test showed that patients in surge 1 presented with significantly lower SBP, lower respiratory rate, and higher oxygen saturation levels on admission compared to surges 2 and 3 (*P* < 0.008). Regarding laboratory parameters, a significant difference was observed in neutrophil (*P*: 0.003) and lymphocyte count (*P* < 0.001), neutrophil to lymphocyte ratio, platelet to lymphocyte ratio, systemic immune-inflammation index (SII), urea, blood urea nitrogen/creatinine ratio, serum potassium, calcium, phosphorus, and magnesium levels of patients between the surges (*P* < 0.001). Additionally, serum erythrocyte sedimentation rate (ESR), C-reactive protein (CRP), alkaline phosphatase (ALP) (*P* < 0.001), hemoglobin (*P*: 0.008), sodium (*P*: 0.009), and aspartate transaminase levels (*P*: 0.010) differed significantly in patients hospitalized across the three surges. Bonferroni post hoc analysis demonstrated that patients in surge 2 were found to have significantly higher platelet to lymphocyte ratio, SII, and urea than the two other surges (*P* < 0.008). Patients from the first wave differed remarkably from those of the two subsequent surges of disease in that they had lower calcium, ESR, and lactate dehydrogenase (LDH) serum levels (*P* < 0.008).

### In-hospital complications and outcomes

**Fig. 1:**
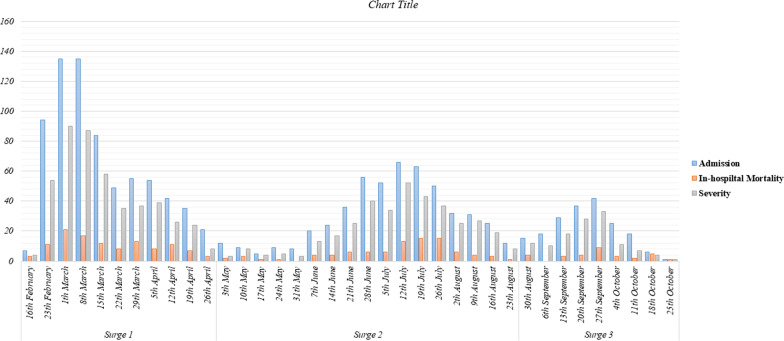
Census of patients admitted with coronavirus disease 2019 (COVID-19) with those who developed severe COVID-19 or were deceased across the first three surges of COVID-19 in Tehran, Iran

**Fig. 2:**
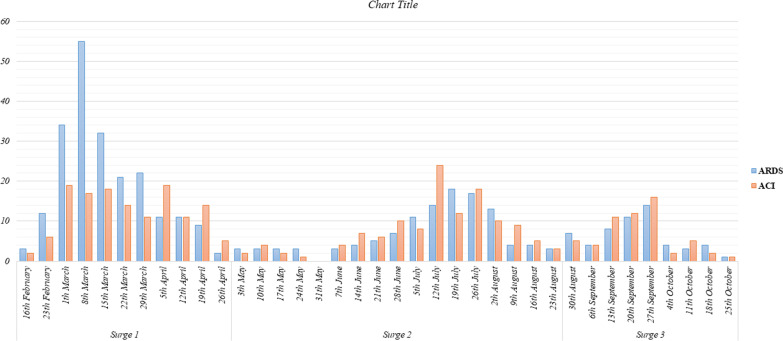
The number of patients who developed acute respiratory distress syndrome (ARDS) or acute cardiac injury (ACI) during the first three surges of coronavirus disease 2019 (COVID-19) in Tehran, Iran

The median length of hospital stay during surges 1, 2, and 3 was 4 days (interquartile range (IQR): 2–7), 6 days (IQR: 3.0–9.0), and 6 days (IQR: 4.0–9.0), respectively (*P* < 0.001). The length of hospital stay was shorter during surge 1 than two other surges (*P* < 0.008). The number of patients requiring invasive ventilation did not differ between the three waves (*P*: 0.149). We presented the number of admitted, severe, and deceased patients based on 1-week intervals in Fig. 1. The mortality rate during surges 1 to 3 was not significantly different, and the number of deceased cases was 114 (17.1%), 91 (18.6%), and 30(18%), respectively (*P* < 0.798). There was no difference in the percentage of patients developing severe COVID-19 with 896 (67.7%) of patients in surge 1, 437 (65.5%) of patients in surge 2, and 349 (71.4%) of hospitalized patients during surge 3 (*P* = 0.094). There were statistically significant differences regarding acute respiratory distress syndrome (ARDS) (*P* < 0.022) and acute cardiac injury (ACI) development (*P*: 0.017) between patients during three surges, which presented based on 1-week intervals in Fig. 2. The number of patients developing acute kidney injury (AKI), acute liver injury (ALI), and multi-organ damage were not significantly different between the surges. After Bonferroni's post hoc analysis application, we observed that during surge 2, patients were less likely to incur ARDS than patients hospitalized during surge 1 and 3 (*P* < 0.008).

## Discussion

The most prominent finding in this study was the difference in age, clinical presentations, length of hospital stay, developing ARDS, and ACI in three different surges of COVID-19 infection. On the other hand, there was no difference regarding disease severity and in-hospital mortality among three different surges. Alpha variant of COVID-19 was active during the first surge in our study. Then, in the end of the second surge from June 22, we faced the Delta variant of the virus resulting in a large number of new cases in July [9].

Older people have been speculated to be more vulnerable to COVID-19 infection and its further complications [10, 11]. The mean age of hospitalized patients in our study was 59.2 ± 16.5. Mean age was lower in our study during the first wave, which is in contrast with the findings of Fluck et al., reporting lower mean age of patients during the second wave [12]. We believe this may be attributed to different study populations and smaller sample sizes in our study, while both studies have included only admitted patients.

The median BMI in our study was 27.4 ± 4.7 kg/m^2^ and was lower than that reported in New York (30 kg/m^2^) [13]. It has been reported that obesity is associated with worse outcomes among COVID-19 patients [14], and a lower BMI in the current study might have been protective. We demonstrated that hypertension, diabetes mellitus, and cardiac disease were the most common comorbidities among patients. Underlying diseases are associated with a higher risk of severe COVID-19 and its complications [15, 16]. In a study comparing two surges of COVID-19 pandemic showed that more than 80% of patients had at least one comorbidity with hypertension being the most common comorbidity similar to our results [17]. Vahidy et al. reported that the number of COVID-19 hospitalized patients with hypertension was significantly higher during surge 1 of the COVID-19 pandemic in Houston, which is in alignment with our results [18].

The prevalence of dyspnea, myalgia and arthralgia, nausea and vomiting, chest pain, and neurological manifestations significantly increased from surge 1 to 3. Gastrointestinal symptoms with more incidence in the second wave were reported in previous observations [19]. Patients admitted during surge 1 had higher oxygen saturation with lower SBP and respiratory rate than surges 2 and 3. The results demonstrate that the non-respiratory presentations of COVID-19 are increasing over time. In contrast, patients admitted during the first surge presented with lower oxygen saturation and higher temperature compared to surge 2 on admission according to Buttenshon et al. study. However, similar to our reports they presented with lower SBP during surge one [17]. The SARS-CoV-2 virus can infect various organs using angiotensin-converting enzyme (ACE2) receptors to enter cells [20]. Therefore, during this pandemic era, extra-pulmonary symptoms should be given more attention by physicians.

Lower than normal absolute lymphocyte count, higher neutrophil to lymphocyte ratio, and elevated than normal CRP, ESR levels are significantly associated with a higher mortality rate in hospitalized COVID-19 patients [21]. Besides, elevated serum levels of creatinine and urea were correlated with hospitalized COVID-19 non-survivors [22]. Our patients differed significantly in neutrophil to lymphocyte ratio, Urea, CRP, and ESR with higher levels of these laboratory markers in surge 2. In contrast, several studies found higher CRP and creatinine levels in patients admitted during surge 1 compared to surge two which were associated with more critical diseases [17, 23].

Patients who developed ACI were reported to be older with more comorbidities such as HTN and DM, lower lymphocyte counts, and higher ALT levels, leukocyte count, and hs-CRP on admission compared to those who did not develop ACI [24]. Similarly, we found that as the number of patients with hypertension rose from surge 1 to surge 3, more patients developed ACI and had lower lymphocyte count than surges 1 and 2. Furthermore, developing ARDS is also associated with elevated cardiac troponin levels and worsened clinical outcomes [25]. We similarly found that the more patients developed ARDS from surge 1 to 3, the more they developed ACI. However, disease severity, in-hospital mortality, and the number of patients receiving invasive mechanical ventilation in our study did not differ across the surges, and the overall in-hospital mortality rate was 17.8% during our study period.

Some previous studies demonstrated a lower disease severity, need for invasive mechanical ventilation, and mortality during the second wave in Europe and the United States [12, 26, 27]. The Netherlands achieved the most considerable reduction in mortality among European countries [26]. While in by Taboada et al. among critically ill patients in Spain, the ICU admission duration and mortality were similar in the three waves [28]. The rate of ICU admission was 16.4% in our study, and patients did not differ in terms of disease severity, ICU admission rate, or mortality among the three surges. This discrepancy may be attributed to different virus strains, vaccination rates, and isolation policies in different countries [12, 26–28].

The strength of the current study is the comparison between three surges of COVID-19 patient's characteristics and outcomes for the first time in Iran as a country with a high burden of COVID-19 pandemic. Several limitations to the current study need to be addressed. First, the present study is an observational study with possible inherent biases. Second, it is a single-center study on the Iranian population, and future multicenter studies with larger sample sizes and different ethnicities are needed.

## Data Availability

The datasets used and/or analyzed during the current study are available from the corresponding author on reasonable request.

## Abbreviations

ACE2: Angiotensin-converting enzyme
ACI: Acute cardiac injury
AKI: Acute kidney injury
ALI: Acute liver injury
ALP: Alkaline phosphatase
ALT: Alanine transaminase
ARDS: Acute respiratory distress syndrome
AST: Aspartate aminotransferase
BMI: Body mass index
BUN: Blood urea nitrogen
CRP: C-reactive protein
DBP: Diastolic blood pressure
ESR: Serum erythrocyte sedimentation rate
hs-cTnI: High sensitive cardiac troponin I
ICU: Intensive care unit
IQR: Interquartile range
LDH: Lactate dehydrogenase
SBP: Systolic blood pressure
SII: Systemic immune-inflammation index
